# Outcomes of a Joint Replacement Surgical Home Model Clinical Pathway

**DOI:** 10.1155/2014/296302

**Published:** 2014-06-12

**Authors:** Avinash Chaurasia, Leslie Garson, Zeev L. Kain, Ran Schwarzkopf

**Affiliations:** ^1^Department of Orthopaedic Surgery, UC Irvine Medical Center, Orange, CA 92868, USA; ^2^Department of Anesthesia and Perioperative Care, UC Irvine Medical Center, Orange, CA 92868, USA

## Abstract

Optimizing perioperative care to provide maximum benefit at minimum cost may be best achieved using a perioperative clinical pathway (PCP). Using our joint replacement surgical home (JSH) model PCP, we examined length of stay (LOS) following total joint arthroplasty (TJA) to evaluate patient care optimization. We reviewed a spectrum of clinical measurements in 190 consecutive patients who underwent TJA. Patients who had surgery earlier in the week and who were earlier cases of the day had a significantly lower LOS than patients whose cases started both later in the week and later in the day. Patients discharged home had significantly lower LOS than those discharged to a secondary care facility. Patients who received regional versus general anesthesia had a significantly lower LOS. Scheduling patients discharged to home and who will likely receive regional anesthesia for the earliest morning slot and earlier in the week may help decrease overall LOS.

## 1. Introduction

Healthcare cost reduction is becoming more than ever the center of every discussion related to innovation in healthcare delivery, especially in the provision of perioperative care. The ability to achieve improvement in quality of care and outcomes while ensuring patient safety and achieving cost reduction is currently the “holy grail” of healthcare delivery. The perioperative period is often a time when many care providers are acting independently, which can easily introduce errors, expenses, and inefficiencies associated with poor coordination of care.

The perioperative clinical pathway (PCP), a concept of a more coordinated surgical care model, is based on coordinating patient care into a team-based initiative and is a paradigm shift that is occurring in the United States [1]. Fleisher et al., Gustafsson et al., and Mythen et al. have reviewed many of the key elements of a PCP to include guidelines for various patients and diseases, as well as surgery specific issues, preoperative testing, perioperative surveillance and therapies, anesthetic considerations, and postoperative and long-term care management [2–4]. This group provided best practice recommendations for various items, including preoperative counseling and recommendations, anesthesia, surgical prep, surgical procedures, fluid management and nutrition, and early mobilization [2, 3, 5].

Lee et al. showed in their literature review of the PCP model that the benefits of a coordinated perioperative system are substantial; namely, there is increased surgical volume and flow (20%–35%), fewer cancellations of surgeries (1%–8%), a reduction in number (23%–55%) and costs (40%–59%) of preoperative cancellations, and a reduction in cost per patient (8%–18%) [1]. An additional benefit of a PCP can be decreased length of stay (LOS) for hospital admission after surgical procedure. Numerous studies have documented decreased LOS with implementation of some or all of the key elements of a PCP [4].

To design a valid model for the joint replacement surgical home (JSH), we must use data based on some of the most common surgical procedures: hip and knee arthroplasties are common and their treatment is expensive. In 2005, the treatment of arthritis in the United States cost over $353 billion [6]. Among the 10 surgical procedures growing the fastest, total knee replacement has the highest aggregated inpatient cost, exceeding $9 billion in 2008 [6]. Tomek et al. found that hospitals that implemented an outpatient preoperative multispecialty evaluation of arthroplasty patients, an inpatient comanagement approach involving anesthesia and orthopaedic surgery and engaging patients in discharge planning prior to admission, all shortened the patients' LOS [7]. PCPs help coordinate the multiple components in patient care and help reduce LOS and costs without compromising patient outcomes [8].

In April of 2012, the joint replacement surgical home was launched at our institution. Following the sponsorship of the departments of anesthesia, orthopaedic surgery, and hospital operations, 5 multidisciplinary teams were established. These teams, which consisted of physicians, nurses, case managers, pharmacists, social workers, IT staff, and others, met weekly for 6 months.

The specific aim of this study is to identify which factors predispose to a longer LOS in a consecutive cohort of JSH patients where total joint arthroplasty (TJA) was performed. We submit that identification of these variables may allow for changes in the design of the JSH to decrease length of stay.

## 2. Methods

### 2.1. Joint Replacement Surgical Home

Based on findings discussed from existing literature our institutional joint replacement surgical home was designed consisting of the following elements.

#### 2.1.1. Preoperative Admission Team

This team includes preoperative evaluation and surgery preparation. This is accomplished by incorporating joint replacement educational classes and providing seamless transition to intraoperative and postoperative care using a nurse navigator. While maintaining a patient centric care philosophy, protocols that were developed for the preoperative team included preoperative evaluation assessment, preoperative testing requirements, preoperative screening protocol, renal risk guidelines, pulmonary risk guidelines, delirium risk guidelines, cardiology consult necessity, dental evaluation, urine analysis,* Staphylococcus aureus* screening and treatment guidelines, and thromboembolic risk assessment.

#### 2.1.2. Intraoperative Care Team

This team coordinates day-of-surgery events from preoperative admission to discharge from the postoperative care unit. They incorporate anesthesia care and regional blocks, efficiency of surgical start time and room turnover time, equipment utilization, and procedural standardization. Protocols that were developed for the intraoperative team included perioperative antibiotic guidelines, normothermic anesthesia goals, and efficiency metrics evaluation including first case start time and case turnover times.

#### 2.1.3. Acute Postoperative Team

This team coordinates the day-of-surgery events from the time of discharge from the postoperative care unit until hospital discharge as well as home healthcare visits and up until the first postoperative office visit. Protocols that were developed for the acute postoperative care team included multimodal pain regiment protocols, pharmacy-led anticoagulation protocol, intensive physical therapy protocols starting from day of surgery, inpatient education, and discharge planning.

#### 2.1.4. Postdischarge Team

This team coordinates patient care from discharge until the 90-day postoperative office visit by optimizing mobility and rehabilitation and preventing perioperative complications. Goals of the team are to avoid readmissions by developing and implementing guidelines for discharge orders, discharge instructions, medication prescriptions, wound care, and follow-up clinic visits.

#### 2.1.5. Quality Measures and Performance Improvement Team

This team is in charge of promoting adherence to evidence-based practice, improving outcomes, increasing patient and staff satisfaction, and utilizing the clinical pathway methodology to manage the JSH. Measures used to evaluate these include quality of life after surgery, decreased hospital LOS, decreased recovery time, decreased rates of perioperative complications, and readmission rates.

The study population of this cohort study consisted of 190 consecutive elective cases of lower extremity total joint arthroplasty that took place at our institution between October 2012 and October 2013. All surgeries were performed by a single surgeon and included total knee and hip replacements. No patients were excluded from this study. A cross-sectional chart review of prospectively collected data was conducted using all TJA cases conducted during the study time period. All identified cases were reviewed and patients' demographic and clinical data were collected.

Prospectively collected data included patient demographics, BMI (calculated using the standard calculation of BMI = weight in kilograms/(height in meters)^2^), hospital length of stay (defined as postoperative number of nights in the hospital after surgery), day of surgery, case order of the day, discharge location, and anesthesia type. Integrity of all data points was confirmed using Decision Support System (hospital-based), electronic medical record (Quest, Allscripts, Chicago, Illinois), and anesthesia information management system (SIS, SISFirst, Alpharetta, GA).

This study was conducted with the approval of our institutional review board (IRB).

### 2.2. Statistical Analysis

In order to analyze the factors that may contribute to LOS, we determined the relationship between each of the variables collected and LOS, separating patients into two groups: THAs and TKAs. For comparison of day of surgery, the LOS of Tuesday patients was compared to the LOS of Thursday patients using a Student's *t*-test, measuring significance by using a critical value of 0.05. This method was also performed to compare all TKAs versus all THAs, married versus nonmarried patients, ASA < 2 versus ASA ≥ 2, age < 65 versus age ≥ 65, PACU arrival time before 2:00 pm versus PACU arrival time after 2:00 pm, cases placed earlier in consecutive order on the day of surgery versus cases placed later in the order on the day of surgery, simple TJA versus conversion and revision TJA, home versus nonhome location of discharge, general versus spinal anesthesia, public insurance versus private insurance, and left-sidedness versus right-sidedness.

A linear regression analysis was also conducted to analyze the *r* ^2^- and Pearson's *r*-value to establish the existence of relationships between BMI and LOS, ASA and LOS, age and LOS, Charlson comorbidity index [9] (extracted from discharge abstract) and LOS, and PACU arrival time and LOS. An |*r* | ≥5 or an *r* ^2^ ≥ 0.5 indicated a correlation between the variables.

## 3. Results

There were a total of 190 patients in our cohort, with 87 THAs and 103 TKAs. The average age of the patients at the time of surgery for THA patients was 63.1 years and for TKA patients was 65.1 years. The average LOS for THA patients was 2.82 days and 2.59 days for TKA patients. The average BMI at the time of operation for THA patients was 29.2 and 30.7 for TKA patients (Table 1).

### 3.1. Univariate Analysis

#### 3.1.1. ASA Score

Patients were pooled into one TJA cohort and then grouped into one of two groups: ASA score ≤ 2 or ASA > 2. There was no statistically significant difference between the ASA ≤ 2 and the ASA > 2 (Table 2).

#### 3.1.2. PACU Arrival Time

Patients were pooled into one TJA cohort and then grouped into one of two groups: arrival to the PACU before 2:00 pm and arrival time at or after 2:00 pm. The PACU arrival time of 2:00 pm was used because that shows surgeries that were started early in the day versus those started later and would not receive a full physical therapy session on the day of surgery. There was a statistically significant difference between the LOS of the early arrival patients and LOS of late arrival patients. Patients who had a later PACU arrival time tended to have a higher LOS (Table 2).

#### 3.1.3. Case Number

Each patient was assigned a number based on their order in the series of conducted on their day of surgery by the single surgeon. There was a statistically significant difference between the LOS of patients who were one of the first two cases of the day and the patients who were third or later cases of the day (Table 2).

#### 3.1.4. Day of Surgery

There was a statistically significant difference between the LOS of the Tuesday THA and the Thursday THA patients and there was also a statistically significant difference between the LOS of the Tuesday TKA and Thursday TKA patients. Thursday THA and TKA patients had higher LOS than their respective Tuesday counterparts. In order to examine that this result is not biased by the complexity of the cases we conducted a second analysis of only the primary TJA cases (Table 2).

When we examined the LOS only on primary TJA cases and removed all complex and revision cases we found that there was no statistically significant difference between the LOS of Tuesday primary THA patients and Thursday primary THA patients. There was also no statistically significant difference between the LOS of Tuesday primary TKA patients and Thursday primary TKA patients. After removing all of the more complicated TJA cases (revision and conversion), Thursday TJA patients do not have a significantly longer LOS than Tuesday LOS patients (Table 2). This result may complement the hypothesis that a standardized perioperative joint replacement surgical home can overcome the difficulties of end of the week care and discharge of primary TJA patients.

#### 3.1.5. Location of Discharge

Patients were put into one of two groups: discharged either to home or to an assisted care facility. There was a statistically significant difference between the LOS of the THA patients discharged home and the THA patients discharged to an assisted care facility. There was also a statistically significant difference between the LOS of the TKA patients discharged home and TKA patients discharged to an assisted care facility. TKA patients discharged home tended to have a shorter LOS (Table 2).

#### 3.1.6. Type of Anesthesia

Patients received either general endotracheal anesthesia or spinal regional anesthesia. There was no statistically significant difference between the LOS of the general anesthesia TKA patients and the spinal anesthesia TKA patients. However, there was a statistically significant difference between the LOS of general anesthesia THA and spinal anesthesia THA patients. THA patients who underwent spinal regional anesthesia tended to have a lower LOS than THA patients under general endotracheal anesthesia (Table 2).

#### 3.1.7. Insurance Type

Patients were divided into groups of having either public health insurance (sponsored by the government at either county, state, or federal level) or private insurance (self-sponsored or sponsored by employer through a private health insurance company).

#### 3.1.8. Surgery Complexity

Patients were classified as either a primary TJA or a more complicated case, such as a conversion TJA, revision TJA, and TJA with hardware removal. There was no statistically significant difference between primary THAs and more complex THAs; however, there was a statistically significant difference between primary TKAs and more complex TKAs (Table 2).

#### 3.1.9. Other Independent Covariables

There was no statistically significant difference between the LOS of all THA patients (mean = 2.82 days) and the LOS of all TKA patients (mean = 2.59 days) (*P* = 0.084).

Relationship status was categorized as either married or nonmarried, with nonmarried patients including single, divorced, and widowed patients. There was no statistically significant difference between the LOS of married THA patients (mean = 2.79 days) and nonmarried THA patients (mean = 2.84 days) (*P* = 0.82) and between LOS of married TKA patients (mean = 2.49 days) and nonmarried TKA patients (mean = 2.72 days) (*P* = 0.116).

BMI was not correlated with LOS for either THA (*r* = −0.0042, *r* ^2^ = 0.0006) or TKA patients (*r* = −0.0121, *r* ^2^ = 0.01) using a linear regression model. Charlson comorbidity index (CCI) was not correlated with LOS for either THA (*r* = 0.01, *r* ^2^ = 0.0002) or TKA patients (*r* = 0.0265, *r* ^2^ = 0.0028) using a linear regression model. Age was taken from the day of birth to the day of surgery; the age of 65 was used to distinguish between younger adults and senior citizens as well as the age cutoff for Medicare. Age was not correlated with LOS for either THA (*r* = 0.0065, *r* ^2^ = 0.0083) or TKA patients (*r* = −0.0062, *r* ^2^ = 0.01) using a linear regression model. Laterality of surgery was simply divided into either left or right. There was no statistically significant difference between the LOS of left hip of THA patients (mean = 2.83 days) and right hip of THA patients (mean = 2.81 days) and between LOS of left knee of TKA patients (mean = 2.59 days) and right knee of TKA patients (mean = 2.59 days).

## 4. Discussion

A thorough background research was conducted as to how to optimize the preoperative, intraoperative, and postoperative experiences for both physician and patient to minimize LOS as part of the establishment of our JSH. In preoperative efforts, to minimize LOS, the greatest active targets have been patient education, preoperative physical therapy, and accounting for the wide diaspora of personal factors that affect LOS [10]. Various healthcare institutions have attempted to implement patient education to decrease patient anxiety and nutrition assessment for morbidly obese patients, both of which have been associated with lower LOS [11]. Additionally, correction of preoperative anemia and initiating physical therapy prior to surgery have been associated with lowering the LOS [11, 12].

In intraoperative efforts to decrease LOS, the focus has been on patients' individual characteristics, such as ASA score, BMI, age, gender, and chronic conditions. Generally speaking, there has been some evidence showing that women, the elderly, and those with more comorbidities have increased LOS and were encouraged to have more resources allocated to them to decrease their LOS [12–15]. Additionally, obesity and Type I and Type II diabetes mellitus were shown to increase LOS, while those with hypertension were not found to have a significantly elevated LOS [16–19]. In our study, BMI has been found to have no correlation to LOS following TJA. In other studies, BMI was positively correlated with LOS, particularly in individuals with a BMI > 30 [17, 20]. Our study also found no correlation between Charlson comorbidity index and LOS, which had not yet been compared in existing literature to examine its relationship to LOS. Our study found ASA to have no significant correlation to LOS, although this has also been shown in other studies as a possible tool to factor into planning a patient's TJA pathway [10, 13, 14, 20].

Neither age nor gender was found to have a significant effect on LOS following TJA in our cohort. While other studies have shown older age to be associated with a longer LOS [10, 14, 20–22] and others showing women to have longer LOS [13], this was not the case in our cohort. This is likely due to the fact that the preoperative joint replacement surgical home (JSH) planning was conducted to provide further resources to the older patients so that their stay in the hospital was not lengthened significantly compared to other TJA patients.

Additionally, the type of insurance was not found to have a significant impact on LOS. There was no significant difference in LOS found between publicly-insured patients and privately-insured patients. This is likely due to the fact that while there are likely differences in insurance decision approval, these differences in approval-making may not be significant enough to impact a patient's mean recovery time.

Marital status was hypothesized to have an effect on LOS, but we found no difference in LOS between patients who were married, divorced, widowed, or single. This had not yet been examined in existing literature in terms of its impact on LOS and recovery following TJA. Our study did find that patients who were discharged home had a shorter LOS than those who were discharged to a skilled nursing facility. This shows that factoring the location of a patient's discharge and their living conditions following surgery can have a dramatic impact on LOS and should be considered as part of a PCP as was done in our institution by implementing our JSH protocols. This has also been shown by others [23].

The use of anesthesia has been shown to be an important factor in a TKA patient's LOS. A British study showed that patients receiving general anesthesia as opposed to regional anesthesia had shorter LOS and better orthostatic function following surgery [19]. One of the main factors that determined a patient's satisfaction with their overall surgical experience was achieving pain relief as expected [12]. We also found anesthesia and its administration to have a role in LOS: THA patients who underwent general, endotracheal anesthesia were associated with a longer LOS than THA patients who had spinal, regional anesthesia. This factor does not seem to be widely reported in the existing literature, though Harsten et al. found that subjects receiving general anesthesia had a shorter LOS and their patients preferred receiving general anesthesia if they had to redo their surgery [24]. This may require further research with a study of higher power and more focus on the anesthesiology side of perioperative care, particularly as to its role in the JSH.

One of the most important factors in recovery was discharge destination following in-house recovery. In another study, patients suffering from anxiety and depression often stayed in the hospital for longer and were more frequently transferred to another healthcare facility, which increased LOS and cost [25]. Additionally, using prehospitalization discharge planning as part of a multimodal clinical pathway was associated with a shorter LOS [26]. In our study we have shown that patients who had been discharged to a skilled nursing facility were shown to have increased LOS. It is evident that taking into consideration where the patient will likely go following their hospital stay will impact LOS.

Many factors play a role as part of a large movement to create an integrated care plan or clinical pathway that could be incorporated into a PCP. In joint replacement surgery, a PCP has been shown to decrease hospital cost and LOS, as well as improving patient satisfaction [10, 23, 25, 26]. One of the most consistent parts of these new programs is to initiate physical therapy on the day of surgery and integrate physical therapy as part of a full seven-day-a-week plan [27–29]. The incorporation of preoperative planning and education, integrating physical therapy into both pre- and postoperative care, and proper pain management have been important factors in creating an effective clinical pathway. It has also been shown that reserving TJA procedures for larger, tertiary-care hospitals provide the best care and shortest LOS for these patients [29].

Our study was also one of the first to see the effect of timing of surgery during a week and during the particular day and its impact on LOS. We found a statistically significant difference between complex patients who often required a stay over a weekend (Thursday TJA patients), when one would imagine a lesser access to resources such as insurance decisions and nursing staff. Even with the current incorporation of twice-a-day physical therapy sessions seven days a week and with a case manager on staff seven days a week, there is still a longer LOS incurred by patients having complex TJA later in the week. We attribute this difference to the lack of contact with insurance providers during the weekend, thus resulting in a delay in discharge location approval for the more complicated and complex cases. Other studies have also shown the inclusion of continuous perioperative supportive care to decrease LOS of TJA patients [14, 27, 28, 30]. When removing the more complicated TJAs (revision and conversion TJA) there was no significant difference established between patients that had surgery on Tuesday versus Thursday. Using these patients as an example, we can see that the JSH protocol allowed the LOS to be the same between patients that have undergone TJA at different times of the week. The JSH then stands to serve as a good model to show how the integration of the 5 teams likely had a key role in creating an optimized experience that was similar for primary TJA patients, regardless of day of surgery.

Our study is also among the first to analyze the effect of PACU arrival time and thus time of surgery and LOS. We found that patients with a later PACU arrival time were associated with longer LOS. Additionally, our study found that surgery complexity and case number have a key impact upon LOS; patients who were simple TJA patients and were one of the first cases of the day had lower LOS. These are key findings that should be considered when predicting ways to minimize LOS as part of a PCP.

There are several limitations to our study. The retrospective nature of the study limits the ability to obtain higher quality data generally associated with prospective studies. In addition, our study was only conducted with 190 patients, and a study with more patients would have higher power, providing more definitive conclusions. Additionally, all the patients were operated on by one surgeon, and a multisurgeon, multi-institutional study could perhaps provide better evidence for what is associated with shorter LOS to be incorporated in the design of a JSH.

The results of our study can be used as part of a sophisticated but simple clinical pathway that should be implemented by a PCP that performs TJA. The existing literature points to the fact that these operations should be best conducted in tertiary-care institutions, with early and thorough patient education, early and consistent physical therapy, and individual planning to minimize LOS [10, 11, 20, 21, 23, 25, 27]. Other factors that have a dramatic impact are management of depression and anxiety and the importance of appropriate pain management in decreasing LOS [12, 24]. Further research into anesthesia (in relation to pain management, ASA score, and operative anesthesia administration) should be conducted to improve this pathway. Additionally, each institution should personalize a JSH to decrease LOS according to the resources and staffing at their disposal.

## 5. Conclusion

Along with a JSH, scheduling simple TJA patients who will likely require spinal, regional anesthesia and be discharged home for morning surgeries earlier in the week may help decrease LOS. We propose that this may expedite these healthier patients so resources can be allocated for those who need more care, so they may also shorten their LOS, as part of the JSH.

## Figures and Tables

**Table 1:** Patient demographics.

Characteristic variable	Total hip arthroplasty mean (standard deviation)	Total knee arthroplasty mean (standard deviation)
Number of patients	87	103
Age at surgery	63.1 years (SD ± 14.3 years)	65.1 years (SD ± 11.3 years)
Length of stay	2.82 days (SD ± 1.02 days)	2.59 days (SD ± 0.69 days)
Body mass index (BMI)	29.2 kg/m^2^ (SD ± 6.13)	30.7 kg/m^2^ (SD ± 5.99)
ASA score	2.72 (SD ± 0.54)	2.82 (SD ± 0.54)

**Table 2:** Summary of conclusive findings.

Independent variable	Mean LOS	*P* value
ASA score		0.631
≤2 (*n* = 50) >2 (*n* = 140)	2.64 days 2.71 days	
Day of surgery (THA)		0.005
Tuesday (*n* = 43) Thursday (*n* = 44)	2.51 days 3.11 days	
Day of surgery (TKA)		0.035
Tuesday (*n* = 57) Thursday (*n* = 46)	2.46 days 2.76 days	
Day of surgery (primary THA)		0.099
Tuesday (*n* = 28) Thursday (*n* = 23)	2.57 days 3.04 days	
Day of surgery (primary TKA)		0.160
Tuesday (*n* = 47) Thursday (*n* = 35)	2.40 days 2.60 days	
PACU arrival time		0.001
Before 1400 (*n* = 92) After 1400 (*n* = 98)	2.49 days 2.60 days	
Case number		0.022
Case 1 or 2 (*n* = 134) Case 3, 4, or 5 (*n* = 57)	2.59 days 2.95 days	
THA location of discharge		0.019
Home (*n* = 40) Secondary care facility (*n* = 47)	2.55 days 3.04 days	
TKA location of discharge		0.0002
Home (*n* = 52) Secondary care facility (*n* = 46)	2.35 days 2.87 days	
THA anesthesia administered		0.0006
General endotracheal (*n* = 32) Spinal regional (*n* = 55)	3.28 days 2.54 days	
TKA anesthesia administered		0.095
General endotracheal (*n* = 28) Spinal regional (*n* = 74)	2.78 days 2.51 days	
THA surgery complexity		0.850
Simple THA (*n* = 55) More complex THA (*n* = 30)	2.78 days 2.80 days	
TKA surgery complexity		0.020
Simple TKA (*n* = 82) More complex TKA (*n* = 22)	2.49 days 4.00 days	
